# Time to conserve… Conservative dentistry?

**DOI:** 10.4103/0972-0707.45246

**Published:** 2008

**Authors:** Velayutham Gopikrishna

**Affiliations:** Editor-In-Chief, Journal of Conservative Dentistry, Department of Conservative Dentistry, Meenakshi Ammal Dental College, Alapakkam Main Road, Maduravoyal, Chennai - 600 095, Tamilnadu, India. E-mail: editor@jcd.org.in

The recently held 9^th^ national FODI and IES Post-Graduate students convention at Goa was a resounding Success. From an academic point of view, it was heartening to see the enthusiastic and overwhelming response in the form of more than 243 papers and posters being presented by the students and staff across the country. However, it is also a time to pause and introspect about the future of our specialty of conservative dentistry and endodontics. Let us look at the following trends to appreciate the status of conservative dentistry in today's perspective. An average postgraduate dentist across the country does hundreds of root canals and is proficient in handling any complex endodontic case. However, the common consensus is that they are not confident enough in doing a ‘class-II direct composite restoration’ or ‘esthetic management’ of anterior teeth. This could be attributed to the examination pattern of most universities. Although we have clear-cut guidelines when it comes to the post-and-core case and the endodontic case, there is a need for consensus in the conservative case. Certain universities follow the metal-inlay exercise, while some have a long case history discussion, while others have any random conservative case management. Look at the recently concluded FODI-IES conference at Goa. In a total of 243 papers and posters presented, more than 75% were endo related. This clearly shows the nationwide tilt toward endodontics. This is also reflected on the number of research projects, dissertations, and publications done by our fraternity which has an undeniable emphasis on endodontics.

I accept the relevance of endodontics in the current clinical scenario where it plays a very significant role. But as guardians of the specialty of conservative dentistry and endodontics, we should also ensure that conservative dentistry also gets its due importance. The need of the hour is;

- Have a common consensus on the type of restorative exam case which should be common across the country and university.
- The federation should specify what should be the minimal clinical criteria a PG student should have completed by way of type of cases and number of cases. This again should be common across the country.

The federation and, more importantly, the members of the fraternity should actively promote the number of research projects and dissertations related to conservative dentistry. The journal of conservative dentistry would provide an ideal platform for dissemination of our research activities. It is a matter of time when our specialty gets bifurcated into individual specialties following the international trend. Hence, it is of paramount importance that we create an atmosphere where the specialties of conservative dentistry not only survive but also form a vibrant potential avenue both academically and clinically.

